# What are the communication guidelines for people with dementia and their carers on the internet and are they evidence based? A systematic review

**DOI:** 10.1177/14713012241292486

**Published:** 2024-10-14

**Authors:** Charlotte Harris, Rebeccah Webber, Gill Livingston, Suzanne Beeke

**Affiliations:** Department of Language and Cognition, 4919 University College London, UK; Division of Psychiatry, 4919 University College London, UK; Department of Language and Cognition, 4919 University College London, UK

**Keywords:** communication, strategies, dementia, carers, internet

## Abstract

**Introduction:** Communication difficulties of people with dementia can negatively impact well-being of them and their carers. There are evidence-based and clinically recommended strategies that can be used to support people with dementia which they are more likely to access on websites than via academic literature. We aimed to search the internet for communication advice for people with dementia and their carers, describe the strategies and compare these to the evidence-base.

**Methods:** After a systematic search of websites offering communication advice to people with dementia and their carers, we described the strategies there, used reflexive thematic analysis to identify the rationale for recommended strategies and compared the strategies to the evidence base. We included websites aimed at people with dementia and their carers published by dementia-related health and social care, or third sector organisations. We compared strategies to those in published systematic reviews and practice guidance from UK health and social care agencies.

**Results:** Our review identified 39 eligible websites, containing 164 individual strategies. These were grouped into 26 strategy types, with nine latent themes developed. These were supporting communication strengths, valuing the interaction, prioritising needs, providing emotional safety, working together, adapting communication for the situation, developing carer communication skills, knowing the individual and focusing on broader meaning.

**Conclusion:** Our review highlights the need for flexible approaches to supporting communication for people with dementia which consider the individual’s needs and preferences, the context of the interaction, and the priority in that moment. We identify the inherent challenges for carers in trying to interpret advice for their own needs.

## Introduction

Being able to communicate with other people is an important part of living well – good communication enabling us to build relationships, maintain a sense of self and have our basic needs met (Alsawy et al., 2017; Nickbakht et al., 2023; Woodward, 2013). Although people living with dementia (people with dementia) retain some, particularly non-verbal, communication skills (Rousseaux et al., 2010), and a desire to maintain a social connection with others (Ellis & Astell, 2017), dementia presents challenges to successful and quality communication including, but not limited to, word-finding difficulties, impaired verbal comprehension and reduced ability to initiate conversations. (Acton et al., 2007; Downs & Collins, 2015; Haak, 2002; Kempler, 1995; Suarez-Gonzalez et al., 2021). This leads to frustration and reduces quality of life for both the people with dementia (Volkmer et al., 2023) and for carers (Georges et al., 2008). The cognitive changes experienced by people with dementia place them at risk of social isolation, and individuals who frequently communicate with them (their communication partners) play an important role in supporting effective communication (Downs & Bowers, 2014; Sabat, 2001). As such, collaborative and supportive communication is key to person-centred dementia care (Kitwood, 1997).

When considering communication strategies, it is helpful to distinguish between message conveyance and social connectedness. O’Rourke et al. (2020) describe person-centred communication as either ‘relational’ or ‘language-based’. They define relational strategies as an approach to communication which is affirming and supportive, and aligns with Kitwood’s ‘positive person work’ (1997). Language-based strategies are, according to these authors, more directly related to the use and understanding of language. Similarly, Alsawy et al. (2017) group strategies as ‘interpersonal’ (such as being respectful; incorporating personal preferences; preserving self-esteem), and ‘practical’ (such as short, simple sentences; verbal reassurance; eye contact). Further, Mok et al. (2021) build on models of conversation in aphasia, a communication difficulty commonly caused by stroke (Kagan et al., 2004), to develop a measure of interaction in dementia focused on skills and strategies of support and participation, categorizing these as ‘Acknowledging Competence’, ‘Revealing Competence’, ‘Interaction’ and ‘Transaction’, where interaction represents the ability to share in a conversation, and transaction represents the ability to convey content. Importantly, when people with dementia have been asked about strategies, themes around ‘sharing emotional connection’ and ‘empowering one’s ability to communicate’ were both present, with the relational aspects feeling most pertinent to them (Alsawy et al., 2020; Volkmer et al., 2023). These distinctions between strategies focusing on social connectedness (relational/interpersonal/interactional) and those which focus on message conveyance (language-based/practical/transactional) have potential for identifying the function or purpose of communication that a strategy may aim to support. This may help communication partners make decisions about which strategies to use, according to the needs and preferences of the people with dementia and the context of the conversation.

A primary focus of communication intervention for people with dementia has been to provide carers (both professional caregivers and family members) with information on how to facilitate communication to maximise the retained skills of the people with dementia (Bourgeois, 2019). Family carers and care home staff may be more likely to access advice and health information from the internet than from published research, being more freely available and aimed at the lay-reader (Jones & Parks, 2021; Kreps et al., 2010). What is currently unknown is how closely web-based communication guidelines relate to the evidence-base around communication in dementia and also to concepts of wellbeing and personhood in dementia care.

This review aims to understand what communication strategies are commonly recommended for people with dementia and their carers. Furthermore, it seeks to compare the advice available on the internet to evidence-based strategies reported in the literature, and to identify a conceptual framework of strategies in the context of theories of communication and well-being. We took the unique approach of completing a systematic review of communication strategies available on websites for people with dementia and their carers, as they are more likely to access this type of content than published research.

## Review questions

1) What strategies are recommended by dementia-focused websites to people with dementia, their carers and care home staff?
2) Do websites refer to the type of communication partner and to the environment or activity in which strategies can be used?
3) What themes capture the concepts or philosophies underpinning these strategies?
4) What is the relationship between published research evidence and guidance on websites which caregivers may be accessing: what are the evidence gaps, and are there strategies which are widely recommended but are without a research evidence base?

## Methods

We conducted a preliminary search of MEDLINE, the Cochrane Database of Systematic Reviews, Prospero and Open Science Framework and no current or underway systematic reviews or scoping reviews on the topic were identified. This review follows the Preferred Reported Items for Systematic Reviews and Meta-Analysis protocols (PRISMA-P) (Moher et al., 2016).

Methods followed an eight-step process for reviewing health information on consumer-oriented websites (Rew et al., 2018). Namely, select a topic (1) and purpose (2), identify search terms and engines (3), develop and apply inclusion/exclusion criteria (4), develop processes to manage search results (5), specify measures of quality (6), and evaluate websites (7) and readability (8).

### Search strategy

• After clearing cache, browser history and location settings, the first author (CH) conducted the search, using incognito settings on search engines where relevant (date of search 28/07/21).
• CH searched the three most commonly used English language search engines (Google, Bing and Yahoo). Grey literature on website search engine use suggests that Google accounts for 91% of search traffic with Bing and Yahoo accounting for a further 6.5% of search traffic (https://www.similarweb.com/engines, June 2023). The terms used for the search were “dementia” AND “communicating OR communication” AND “strategies OR tips OR advice”.
• Only 5% of search engine users look beyond the first page of results (https://www.brafton.com) and 88% of people click on the first 10 results only, with reductions from first result (28.5%) to 10^th^ (2.5%) (https://www.searchenginejournal.com). Therefore we used only the first 50 results for each search engine to ensure a process identify sites most often accessed by members of the public, and therefore should achieve data saturation.

### Inclusion criteria for websites

• Created by dementia-related charities, health/social care organisations or not-for-profit advocacy organisations
• Provide information for people with dementia and their carers (family and staff) about dementia-related communication difficulties at any stage of dementia, with at least one section dedicated to communication strategies
• Published in English
• Freely available to the public

### Exclusion criteria for websites

• Promoted or sponsored links within search engine results
• Blogs created by individuals Newspaper or magazine articles
• Peer reviewed journal articles
• Stand-alone audio-visual or graphic resources

### Data extraction

**Figure 1.**
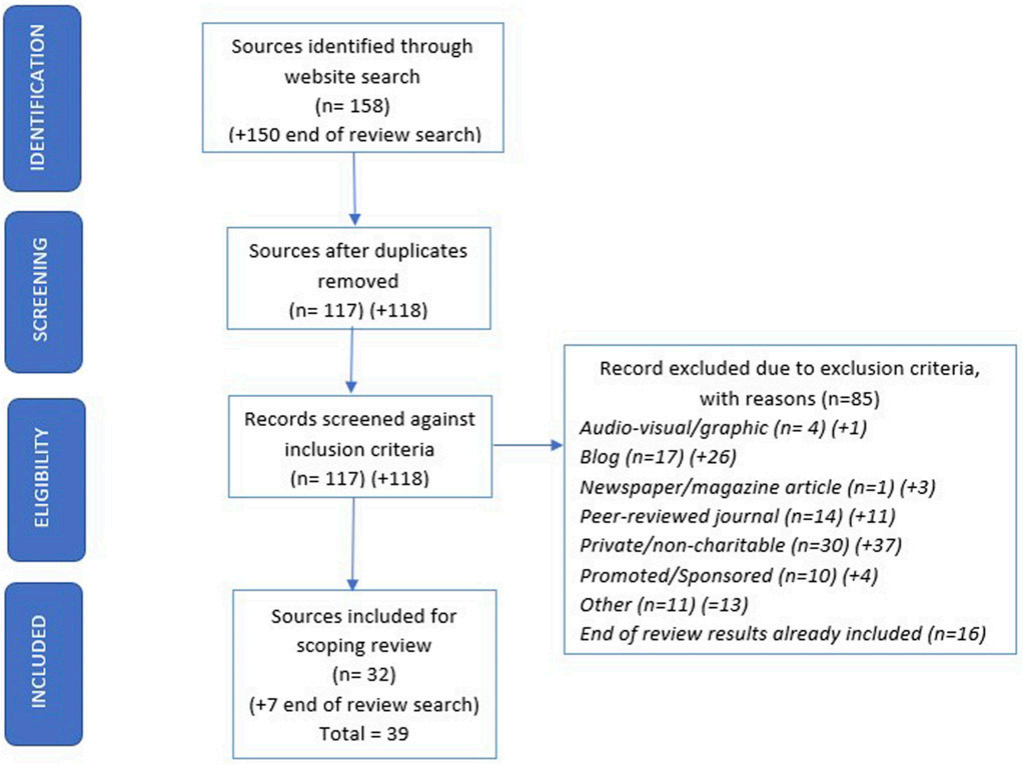
PRISMA-P Flow Diagram (including end of review searches, with additional results indicated by +).

• CH recorded the date and took a screenshot of the search results and saved as a pdf file on the day of the search. See PRISMA-ScR flow diagram (Figure 1).
• CH exported the first 50 results into a Microsoft Excel spreadsheet. After removal of duplicates, these were examined by her and RW against the inclusion and exclusion criteria. They recorded reasons for exclusion and resolved disagreements through discussion.
• CH and RW extracted data into a spreadsheet, including the website URL and country of origin and covering quality criteria (Silberg et al. (1997): a) author/source and credentials (authorship), b) links to evidence sources and references (attribution) c) website ownership and description of purpose (disclosure) d) year of copyright and most recent update (currency).
• CH and RW also extracted verbatim text from the web-page and from links on communication strategy pages within each website and included these but did not follow links to external sites.
• CH used the DISCERN instrument for evaluating written consumer health information to evaluate the quality of information on (Charnock et al., 1999). RW independently completed 50% of the DISCERN evaluations. As recommended they used a consensus approach to the overall rating for each source. Where there was disagreement, they discussed scoring rationale until they reached agreement.
• CH and RW assessed readability by using the Flesch-Kincaid readability score (Flesch, 1948), where 12–15 year olds generally understand text with a score between 60-80.
• CH and RW assessed whether the perspective of the person living with dementia was included.
• CH and RW repeated the initial website search prior to final write-up to check for additional sources (date of search 06/04/23).

### Data analysis and presentation

• CH calculated the frequency of strategy recommendations across sites (how many websites contained a particular strategy) as well as frequency of mention (how many times a strategy was mentioned across all websites). She then combined similar strategies (e.g. ‘Slow down your speech’ and ‘Speak slowly’ were both coded as ‘Speak slowly’). These combinations were reviewed by a PPI group of four people with lived experience of dementia. The PPI groups were research network volunteer networks recruited to monitor the study and contribute their views, as such ethical approval was not required. The group considered whether they felt the grouped strategies were the same and whether there was any overlap between them. After this some strategies were further combined.
• In order to analyse the full content, any additional piece of advice was coded as a strategy. The combined list of strategies was reviewed for any duplication in explicit meaning.
• Next, the PPI group identified whether they used the strategies and find them to be effective, to gain a sense of how the findings resonate for people with dementia and carers. The group were asked to rate strategies as green (a strategy they have used and believe to be helpful), orange (a strategy they are not familiar with or are unsure whether it would be helpful or not), or red (a strategy they would not use, or believe to be unhelpful). They were asked to reach a consensus on these ratings and to make comments to explain their ratings. In some cases, we agreed to amend a strategy name so that it was easier to relate to.

### Comparing to the evidence-base

In order to compare the extracted strategies to the evidence base, CH conducted a search for systematic reviews of communication strategy effectiveness using the databases PROQUEST, Medline, PsycInfo, Amed, Web of Science, Cochrane, and Prospero. This generated five relevant systematic reviews (Alsawy et al., 2017; Egan et al., 2010; Kindell et al., 2017; Swan et al., 2018; Vasse et al., 2010). Practice guidance from NICE and SCIE was also reviewed (National Collaborating Centre for Mental Health (UK), 2007, National Institute for Health and Care Excellence, 2018, Social Care Institute for Excellence, 2013). The five systematic review papers used different ratings systems to evaluate the evidence (see Appendix D for details).

### Reflexive thematic analysis

CH completed reflexive thematic analysis to identify why strategies are used (see Appendix B for a description of the two-stage analysis process). CH coded the extracted strategies, identifying an item of verbatim website data sitting under each strategy to code together with the strategy – this was important as strategies often had more than one purpose and therefore multiple codes. Thematic analysis of website strategies was iterative and reflexive following Braun and Clarke’s (2006) six phase approach (familiarisation; coding; generating initial themes; reviewing and developing themes; refining defining and naming themes; and writing up) with further guidance from Braun and Clarke (2022).

CH maintained a reflective journal and considered her own positionality as a white, English-speaking speech and language therapist with over 15 years’ experience of supporting people with dementia. She also has a parent with dementia. CH developed codes inductively starting with descriptive/observational labelling and moving towards exploration of code labels. These were then grouped into higher order themes which CH and co-authors SB and GL discussed and refined.

## Results

### Website characteristics and quality measures

The initial search (see Figure 1) identified 32 eligible websites for data extraction and analysis, with a further 7 eligible websites identified in the updated search. Included websites originated from the UK (14), USA (13), Canada (5), Australia (4), New Zealand (1), Ireland (1) and India (1). Table 1 presents a summary of website evaluations, reporting quality of health information, readability scores and whether the perspective of the person living with dementia was included (see Appendix A for a full list of websites and evaluations).

**Table 1.** Summary of website evaluations.

Silberg et al. (1997)	Authorship	17/39 websites (44%) gave author details
	Attribution	7/39 websites (18%) cited or gave direct links to evidence sources
	Disclosure	39/39 websites (100%) gave information about website ownership and a description of purpose
	Currency	34/39 websites (87%) have the date of creation or last update. 22/39 (65%) had been reviewed or updated since the latest NICE guideline for dementia (NICE, 2018) was produced
Charnock et al. (1999)	Discern evaluation	20/39 websites (51%) had an overall Discern score of 3/5 (i.e fair) or above. One site achieved an overall score of 5/5
Flesch (1948)	Flesch-kincaid readability score	20/39 websites (51%) had a readability score over 60, indicating a level equivalent to ‘plain English’
Inclusion of perspective of people with dementia		6/39 (15%) websites explicitly included the perspective of people with dementia, either by including quotes from people with dementia, or being wholly or partially written by them

NICE = national institute for health and care excellence.

### Strategies recommended across websites

Initially, we identified 173 different strategies across 32 sites. After review with the PPI group, the list was reduced to 164 strategies which CH combined to create 26 groups according to similarity in strategy type – for example, strategies related to question use were grouped as ‘Thinking about how you use questions’. Each group contained between 3 and 15 strategies (see Table 2).

**Table 2.** Strategy Types and number of strategies within each group.

Strategy type	N	Strategy type	N
Using and observing non-verbal communication	15	Preparing for conversation	6
Being kind, friendly and natural	10	Remembering your own needs	6
Keeping words and sentences simple and relevant	8	Being open about communication difficulties	5
Respect and dignity	8	Ensuring a relaxed, calm environment	5
Considering the whole person and all their needs/challenges	7	Listening carefully and paying attention	5
Getting to know the person with dementia’s communication strengths and supporting them when needed	7	Knowing about the person as an individual, their life history and background	5
Giving time in a conversation	7	Using activities and objects to support interaction	5
Helping the person with dementia find different ways to express what they want to say	7	Recognising that criticism and over-correction aren’t helpful	5
Supporting the person with dementia to understand what you are saying	7	Supporting the person with dementia to understand what you are doing together	5
Starting and ending conversations thoughtfully	7	Managing difficult behaviour calmly and sensitively	4
Thinking about how you use questions	7	Using touch respectfully	4
Considering the person with dementia’s underlying feelings	6	Involving the person with dementia in conversations	4
Knowing about dementia and understanding the challenges	6	Speaking slowly and clearly	3

N = number of strategies in the group.

### Reference to the type of communication partner or to the environment or activity in which strategies can be used

Most websites (37/39, 95%) were aimed at carers generally, either family caregivers or paid carers (see Appendix A for details of websites including reference codes). Of these, two sites had separate sections for family and professional carers (B11 and B33). One website (B41) is aimed at churchgoers, with guidance on how to communicate with people with dementia who may be attending church, and one website (Y54) is aimed at people with dementia themselves with separate sections for social interaction and communicating with professionals. Only 6/39 websites (15%) give specific suggestions for stimulating activities, and for how communication strategies could be used during these activities, or how the activities could promote communication.

### The relationship between published research evidence and guidance on websites which caregivers may be accessing

There was at least one evidence source for 100/164 strategies. Of these 100 strategies, 48 (48%) had rated evidence strength. 10/48 strategies had the highest rated evidence (SORT A, Level II or above, QATSDD 80% or above (see Table 3). A further 22/100 (22%) strategies were recommended in the NICE Guidelines (NICE, 2018) suggesting robustness of evidence, despite not being amongst the highest rated evidence seen. Of the 64/164 (39%) strategies that were not reflected in the evidence base, 42 (66%) were rated as green (helpful) by the PPI group. In total 120/164 strategies were rated as green by the PPI group (73%) and 11/164 (7%) were rated as red (i.e. not helpful), 6 of which were reflected in the evidence-base as effective strategies. Eleven of the 64 strategies not reflected in the evidence base (17%) appeared on more than 10 websites and were mentioned 15 times or more across all websites (Table 3). There was only one communication strategy identified within the evidence which did not appear on any website, which was that carers could try ‘Doing things yourself’ (Alsawy et al., 2017) that is complete tasks or make decisions themselves without involving the people with dementia to avoid potential communication challenges. Appendix D gives full details of how strategies map onto the evidence, and the quality of evidence in each case.

**Table 3.** Strategies, strength of evidence, frequency of website mention, PPI rating and related theme.

Strategy (strategy type)	Strength of evidence and source	Frequency of mention on websites	PPI rating (green, orange, red)	Related theme numbers
With most robust evidence (ordered by frequency of mention on websites)				
**Avoid arguing** (Criticism and over-correction aren’t helpful)	QATSDD over 80% (Alsawy et al., 2017^1^)	19	Green	4, 7, 8, 9
**Support PLWD to reminisce without testing them** (Thinking about how you use questions)	SORT A (Vasse et al., 2010)	18	Green	2, 5, 8
**Use a calm friendly tone of voice** (Using and observing non-verbal communication)	QATSDD over 80% (Alsawy et al., 2017)	17	Green	4, 7
**Be honest without being hurtful** (respect and dignity)	QATSDD over 80% (Alsawy et al., 2017)	9	Green	4, 8, 9
**Stick to one topic at a time** (Keeping words and sentences simple and relevant)	SORT A (Egan et al., 2010; Vasse et al., 2010) and Level II (Swann et al., 2018)	4	Green	1, 5, 7
**Consider the culture and background of the PLWD** (Knowing about the person as an individual, their life history and background)	QATSDD over 80% (Alsawy et al., 2017)	4	Green	8
**Give friendship and support** (Being kind, friendly and natural)	QATSDD over 80% (Alsawy et al., 2017)	4	Green	2, 4, 8
**Initiate conversations** (preparing for conversation)	SORT A (Vasse et al., 2010)	4	Red	5, 7
**Learn to recognise a persons’ non-verbal messages** (Using and observing non-verbal communication)	SORT A (Vasse et al., 2010)	3	Green	7, 9
**Be positive** (Criticism and over-correction aren’t helpful)	QATSDD over 80% (Alsawy et al., 2017)	3	Orange	4, 7
Mentioned 15 times or more across websites but not found in the evidence (ordered by frequency of mention on websites)				
**Be relaxed and calm** (Being kind, friendly and natural)	Not in evidence sources	29	Green	3, 4
**Speak clearly** (speaking slowly and clearly)	Not in evidence sources	27	Green	1, 7
**Be patient** (Giving time in a conversation)	Not in evidence sources	25	Green	3, 4, 5, 9
**Use conversation or non-verbal interaction to connect and show you care** (Being kind, friendly and natural)	Not in evidence sources	22	Green	2, 4, 5, 7, 8
**Sit or stand where the PLWD can easily focus on you and not feel intimidated** (starting and ending conversations)	Not in evidence sources	22	Green	2, 4, 5
**Don’t ask lots of direct testing questions at a time** (Thinking about how you use questions)	Not in evidence sources	21	Green	1, 4, 5, 7, 9
**Introduce yourself** (starting and ending conversations)	Not in evidence sources	19	Green	1, 4, 7, 8
**Don’t talk down to the PLWD** (respect and dignity)	Not in evidence sources	17	Green	4, 8
**Get their attention first** (Listening carefully and paying attention)	Not in evidence sources	17	Green	1, 2, 5
**Use simple words** (Keeping words and sentences simple and relevant)	Not in evidence sources	16	Red	1, 7
**Be creative and flexible** (Using activities and objects to support interactions)	Not in evidence sources	15	Yellow	4, 5, 6, 7, 8, 9

PLWD = people living with dementia; SORT = strength of recommendation Taxonomy; QATSDD = Quality Assessment tool for studies with diverse designs; Levels = NHMRC ratings; PPI = Patient and public involvement group.

### Themes capturing the concepts, philosophies and reasons underpinning strategies

The reflexive thematic analysis highlighted the purpose of different strategies. Nine themes were identified and these have broad representation across the 26 strategy groups. Appendix C shows each theme, with underlying codes, and the representation of that theme across strategy types (shown by the number of groups containing coded strategies). One strategy type (‘Using and observing non-verbal communication’) was coded in all nine themes. All themes contain examples from the most frequently mentioned, but not evidenced, strategies and all but two themes include some of the most robustly evidenced strategies (see Table 3). Each theme can best be described with the use of examples from the strategy data:

1. SUPPORTING COMMUNICATION STRENGTHS – One of the most robustly evidenced strategies in this theme is “Stick to one topic at a time”. Taking a strength-based approach, this theme acknowledges that the people with dementia retains communicative abilities and planning for communication should maximise these. This could be about taking practical steps such as “Make sure they are wearing their glasses”; “Try to ensure people are in familiar and comfortable surroundings”. There are also strategies around how the communication partner manages the interaction, specifically “Give people time to think and respond”. An important component is the idea of assuming that communication is possible, that the people with dementia can engage in a conversation and express their thoughts and feelings, if the appropriate support is given; expressed as “Don’t make assumptions about ability to communicate”.
2. VALUING THE INTERACTION – “Give friendship and support” is a well-evidenced strategy in this theme. It suggests acknowledging humour and the use of laughter to create connection - “Find shared humour, laugh together where you can”. There is a strong sense of being able to enjoy and appreciate time with someone, irrespective of whether a clear message or meaning has been conveyed so “Give friendship and support”. As well as valuing how the people with dementia communicates with them, carers should also endeavour to demonstrate, through communication, that they value the person, to “Use conversation or non-verbal interaction to connect and show you care”.
3. PRIORITISING NEEDS – No strategies within this theme had robust evidence. There is an inherent contradiction in this theme, as the two codes demonstrate the struggle between carers being advised to look after their own needs (“Walk away if you need a break”) and carers being expected to put the people with dementia first by creating a calm atmosphere (“Be relaxed and calm”).
4. PROVIDING EMOTIONAL SAFETY – Evidenced strategies in this theme include “Use a calm, friendly tone of voice” and “Be positive”. There is some overlap with Valuing the Interaction (theme 2) but focuses on interacting with respect and kindness, avoiding conflict and helping the person to feel comfortable and reassured. Strategies around how to use honesty (“It isn’t helpful to mislead people living with dementia”) while validating the person’s reality are included here (“Accept their reality and join them in it”). Recognising the ability of the people with dementia to feel emotions which they may not be able to express verbally is also important (“Look for the feelings behind the words being said”). Avoidance of conflict (“Avoid criticizing or correcting”) and communicating in a respectful way are integral to this theme (“Don’t talk down to the people with dementia”). This theme occurs in 24/26 of the strategy types (see Appendix C).
5. WORKING TOGETHER – The most robustly evidenced strategies within this theme are “Support people with dementia to reminisce without testing them” and “Initiate conversations”’. There is a sense of the collaboration that can exist between the people with dementia and the communication partner, a sense of shared power and responsibility (“Take time to check your understanding of what they have said and the success of the communication”; “Focus on their strengths rather than what they find difficult”), and of inclusion (“Sit or stand where the people with dementia can easily focus on you and not feel intimidated”; “Avoid interruptions”). There is acknowledgement that despite all best efforts, sometimes meaning will not be effectively conveyed (“Accept that the people with dementia will make mistakes with their words sometimes”). This theme cuts across a large number of strategies, with 25/26 strategy types (see Appendix C) occurring.
6. ADAPTING COMMUNICATION FOR THE SITUATION - No strategies within this theme were reflected in the most robust evidence sources. This theme reflects the need for flexibility in the moment, which might be about recognizing when something is not working and being ready to try a different approach (“Be prepared to repeat, rephrase and keep trying several times”; “If the person with dementia doesn’t understand you try a different approach”). Context is important to the choice of strategy and this is reflected in a flexible approach to communication, as demonstrated by the contradictory advice given around use of questions (“Use close questions”; “Use open questions”).
7. DEVELOPING CARER COMMUNICATION SKILLS – One of the most robustly evidenced strategies within this theme was “Learn to recognise the person’s non-verbal messages”. The importance of carers and communication partners working at communication, to reflect on their style (“Learn what might be helpful from previous interactions”) and adapting it over time is clear. Strategies acknowledge that this may be difficult but is important, suggesting “Look for shared meaning in what people with dementia say or do even if it seems confusing”. The responsibility for adapting is placed with the carer to “Learn about dementia and the changes that occur”, as is the need to have a “Ensure a consistent approach to communication” and to put effort into getting communication right, to “Think about what you are going to say and how”.
8. KNOWING THE INDIVIDUAL - HISTORY AND PRESENT – This theme links to the well-evidenced strategy to “Consider the culture and background of the person”. Knowledge of the individual is key. This is partly about acknowledging the person’s life history, to “Keep the person at the centre and remember what you know about them”. It is also about recognising their perspective and feelings (“Acknowledge their feelings and support them to express them”) and knowing about the individual’s particular communication approach (“Encourage communication in any way that works for the individual”). It also incorporates the need for empathy (“Think about how it might feel if you struggled to communicate”).
9. FOCUSING ON BROADER MEANING – The most robustly evidenced strategies related to this theme include “Avoid arguing” and “Be honest without being hurtful”. This theme was developed from a combination of ideas around finding meaning and focusing on an overall message rather than on details. This includes ideas around letting go of the need for perfection to “Accept that the people with dementia will make mistakes with their words sometimes” and looking for meaning in feelings even if language is not accurate, so “Look for the feelings behind the words being said”. This involves a stronger reliance on non-verbal communication (“Learn to recognise a person’s non-verbal messages”) and the need to look beyond words to find meaning (“Listen carefully and actively to what the people with dementia says”).

## Discussion

This review of internet-based communication advice found 164 strategies across 39 websites from seven countries. The strategies were grouped into 26 strategy types. 100 strategies were represented by evidence presented in systematic reviews and best-practice guidance, although many that were not represented in the evidence-base were deemed to be useful by people with lived experience and/or occurred with high frequency on websites. A reflexive thematic analysis identified nine themes relating to the purpose of the strategies: supporting communication strengths, valuing the interaction, prioritising needs, providing emotional safety, working together, adapting communication for the situation, developing carer communication skills, knowing the individual and focusing on broader meaning.

### Relationship of themes and conceptual frameworks

The themes give a strong sense of the value of emotional connection during interactions, suggesting this is as important as the accurate delivery of a message when defining successful communication. Providing Emotional Safety (4) and Working Together (5) are particularly well represented across all strategy groups, demonstrating the overarching value of kindness, respect and collaboration for a successful interaction. The strategy group ‘Using and Observing Non-Verbal Communication’ contains strategies which are coded under each of the nine themes. This demonstrates the broad role of non-verbal communication, both as a means of supporting the delivery of a message, but also as a way of demonstrating empathy, respect, the pleasure of interaction and recognition of individuality.

**Figure 2.**
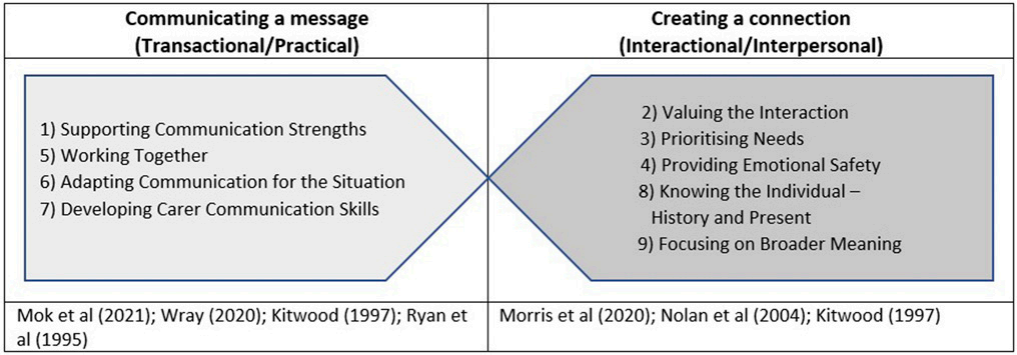
The relationship of strategy themes to conceptual frameworks.

The themes identified are consistent with a distinction between transactional and interactional communication (Mok et al., 2021) and Alsawy’s (2017) categorization of strategies into practical and interpersonal. There are four themes with a focus on communicating a message: Supporting Communication Strengths (1), Developing Carer Communication Skills (7), Adapting Communication for the Situation (6) and Working Together (5), all of which, while supporting well-being and self-esteem, are essentially focused on increasing the likelihood of a ‘successful’ transaction of information through collaboration and flexibility. These ideas are represented in models of successful communication in dementia (Morris et al., 2020; Ryan et al., 1995; Wray, 2020) which talk about maximizing skills, considering the environment and modifying output, with the aim of sharing information successfully. Sitting alongside these, five themes focus on creating a connection: Valuing the Interaction (2) and Providing Emotional Safety (4), Knowing the Person (8), Prioritising Needs (3) and Focusing on Broader Meaning (9). These themes represent validation, acceptance, celebration and warmth, all of which are described by Kitwood (1997) as enhancers of personhood. There are also links to Nolan’s (2004) ‘senses’ or indicators of good relationship-centred care, which highlights a sense of emotional security, belonging (through knowing each other) and, crucially, considering the psychological needs of both partners in the communication. Morris et al. (2020) also highlight the importance of reciprocity and understanding each other’s perspective. Figure 2 shows the 9 themes and how they relate to the two categories described.

It is important to note that the distinction between transactional and interactional strategies is not completely dichotomous and that most themes contain some elements of both ‘communicating a message’ and ‘creating a connection’. For example, Working Together (5) centres the people with dementia and acknowledges personhood and autonomy through collaboration (Kitwood, 1997) but the primary rationale for strategy use is to improve transactional aspects of the communication. Similarly, Knowing the Individual – History and Present (8) is primarily about honouring individuality and understanding the people with dementia’s perspective and reality, but also encompasses understanding their individual communication style to promote successful message conveyance. This is indicative of how interlinked communication skills are with person-centred care and approaches to well-being, and how the use of supportive communication is as much about the functional need to exchange information as it is about the need to build confidence and motivation to communicate while maintaining a relational bond. This conceptual link, and the mutual influence of transactional and interactional approaches, has been reflected in the views of people with dementia (Alsawy et al., 2017). Their preference for interactional strategies perhaps suggests that ‘creating a connection’ forms the foundation for supportive communication, which language-based, transactional strategies can build upon.

### Demands associated with website advice

The reflexivity of the analytic process highlighted concerns for CH around the directive, and at times unrealistic, way some strategies are presented on websites, and also the impact of her own positionality. This quote from her reflexive journal expresses her awareness of personal and clinical influences:

“I inevitably think about people I’ve worked with and how useful a strategy may/may not be. I think of Dad, and how he might respond to how something has been worded. My clinical experiences mean I am less keen on ‘black and white’ sets of strategies – I know that in the real world not everything works for everyone and this clouds how I see very directive advice. Perhaps I think about whether or not I would give that advice myself. I need to remain open-minded to all the strategies and ideas I see presented.”

Taking the perspective of the carer, some strategies feel demanding, with high expectations to act in a way which may be hard to maintain. This is of course a subjective viewpoint, although the thoughts were shared by the PPI group, whose input was important given the limited perspective of people with dementia on the websites themselves. Often, advice is presented in a very directive way (‘Do X’ or ‘Do not do Y’) and feels overly simplistic for the realities of moment-by-moment communication and its complexity. Examples of this include, “Retain a sense of humour in difficult situations” “Don’t be personally offended if the person who has dementia becomes paranoid or accusatory” and “Remember it’s not you who is important but the person with dementia” In a similar vein, the PPI group rated “Initiate conversations” as orange (a strategy they are not familiar with or are unsure whether it would be helpful or not) because they interpreted this strategy as meaning the carer should always be the one to initiate, which they did not agree with. The large number of varied strategies place huge demands on carers to adapt, reflect and change lifelong communication habits, and to continue adapting as the people with dementia’s needs change. It risks ‘over-accommodation’ of communication style by the carer, in trying to implement too many strategies indiscriminately, which can elicit negative responses from a person with a communication difficulty, as it may be viewed as patronising (Simons-Mackie, 2018).

There are contradictions across the strategy data, with clear contradiction sometimes in evidence. Carers are told to “Follow their lead” (site G23) but also to “Lead the conversation and allow the person to join in” (site B3). They are also advised to “Be aware of the space between you and the person, they may not be comfortable with you in their “whisper zone” or “personal space”” (Site G31) while also being told to “Get close enough so they can see your facial expressions and any gestures you may use” (site G10). In order to deal with such contradictions, a carer will need to judge the context of the situation, know the individual, and be able to reflect when things go wrong in an interaction, in order to adapt how they communicate accordingly. Another interesting contradiction in the advice is around the use of questions and whether these should be open or closed. The case for closed questions is presented strongly across 18 of the 39 sites, for example “Avoid asking open-ended questions” (site G39) or “Those with yes or no answers work best” (site B50). Only two websites suggest the value of sometimes using open questions, with this being context or goal dependent “Use open ended questions when you want to open up conversation” (sites B18 and G31). Again, this reflects the sense that flexibility and adaptability are important, rather than suggesting carers should stick to a set of rules they must never break.

### Evidence for strategies

There was a clear lack of evidence for widely recommended strategies, with only 61% (100/164) of identified strategies being represented in systematic reviews of communication strategies or in national guidance. Only 6 of 39 websites included advice directly from the viewpoint of people with dementia. However, our PPI group were able to reflect on the strategies and indicate that many of those not reflected in the evidence nevertheless felt useful (120 strategies in total, 42 of which were not reflected in the evidence-base). The lack of published evidence does not suggest that strategies on websites are unhelpful approaches, but perhaps that they may be grounded in anecdotal guidance. In fact, some of the best evidenced strategies share a strategy group with strategies not mentioned in the evidence but with broad website coverage (Table 3). Strategy groups containing both well-evidenced and frequently mentioned but not evidenced strategies are: ‘Thinking about how you use questions’, *‘*Using and observing non-verbal communication’, ‘Respect and dignity’*,* and *‘*Keeping words and sentences simple and relevant’. This suggests that while strategies represented in the evidence-base may generally be viewed with more confidence, there are a number of strategies anecdotally felt to be useful but missing from the current evidence-base. Despite the lack of grounding in evidence for some strategies, as a whole the strategies map across well-established concepts and theories of dementia care. It is also important to acknowledge that each person’s unique context and individual experience is likely to impact on how they rate a strategy’s usefulness.

This study is limited by challenges associated with internet searching, with a large number of results potentially fluctuating daily, although this was somewhat mitigated by the searches being re-run at the end of the study (which only uncovered an additional 7 sites). In addition, by focusing the search on websites seen as more credible that is those published by health or social care organisations and dementia focused charities, this excluded personal blogs which many carers may gravitate towards for a more personalised (and potentially more accessible) account. We also did not include audio-visual sources, such as YouTube videos, which are increasingly popular. A review of graphic and video resources would be a useful addition to this field. The knowledge identified through this review will benefit from the addition of further research to identify communication approaches that work best in different contexts involving people with dementia and how best to support carers to maximise effective communication. Further observational investigation of everyday communication in context will help to develop understanding of the value and role of both transactional and interactional aspects, as well as increasing the available evidence base for communication strategies that help people with dementia.

## Conclusion

This review finds a large number (164) and wide variety of communication strategies recommended to carers of people with dementia on web-based resources. The themes identified across these strategies bring together ideas from established models of well-being and communication, and demonstrate the inter-dependent nature of these ideas. The purpose of the strategies identified either relates to communicating a message, or to creating a connection. Many overlap, which demonstrates the importance of both achieving the message and creating emotional safety. The need to establish mutual understanding and the need for social connectedness both exist, and there is a demand for carers to be adaptable to fluctuating context and the changing needs of the people with dementia. It is challenging to expect people with dementia or their communication partners to prioritise one communicative need over the other, particularly under difficult and changing circumstances, and they may need support from professionals to identify when and how to adapt their focus, and consequently their strategy choice. An accessible resource, based on strategy principles, which develops awareness of the need for flexible approaches to communication challenges would be a valuable resource to develop in the future.

## Supplemental Material

Supplemental Material - What are the communication guidelines for people with dementia and their carers on the internet and are they evidence based? A systematic review Supplemental What are the communication guidelines for people with dementia and their carers on the internet and are they evidence based? A systematic review by Charlotte Harris, Rebeccah Webber, Gill Livingston, and Suzanne Beeke in Dementia
