# Supplementary material for: What are the communication guidelines for people with dementia and their carers on the internet and are they evidence based? A systematic review
Source: Dementia (London). 2024 Oct 14;24(3):577–94. doi: 10.1177/14713012241292486 (PMC11915767; doi:10.1177/14713012241292486)
Supplement: Supplemental Material - What are the communication guidelines for people with dementia and their carers on the internet and are they evidence based? A systematic review [file sj-pdf-1-dem-10.1177_14713012241292486.pdf]

Appendix A

Website Details and Evaluations

Website Reference	Website Host Address	Country of Origin	Authorship	Attribution	Disclosure	Currency	Overall Discern Score	Flesch-Kincaid Score	PLWD perspective included
B33	https://www.uofazcenteronaging.com	USA	Yes	Yes	Government resource	2018	1	52.5 (74.5 for family carer pages)	No
B11	http://www.demtalk.org.uk	UK	Yes	Yes	Not for profit resource	2011	4	65.8	No
B3	https://www.alzheimers.org.uk	UK	Yes	Available on request	Charity resource	2020	4	63.9	No
G23.2	https://myhealth.alberta.ca	Canada	Yes	No	Public health resource	2022	4	69.2	No
G22.2	https://www.alzscot.org	UK/Scotland	Yes	No	Charity resource	2022	3	68.1	No
B28	https://dementiacarenotes.in	India	Yes	No	Not-for-profit resource	2021	5	63.5	No
G21	https://ocfch.org	USA	Yes	No	Charitable network	2021	3	75	No
G23	https://www.seniorsmatter.com	USA	Yes	No	Non-profit organisation	2021	2	54.7	No
B34	https://www.alzheimers.net	USA	Yes	No	Online advocacy network	2021	2	52.5	No
G32	https://www.brightfocus.org	USA	Yes	No	Non-profit organisation	2020	3	65.8	No
B45	https://patient.info	UK	Yes	No	Health service resource	2020	2	56.8	Yes
G31	https://www.islandhealth.ca	Canada	Yes	No	Public health resource	2018	4	54.4	No
G18.2	https://www.nia.nih.gov	USA	Yes	No	Public health resource	2017	3	73.7	No

G45	https://dementia.nz	New Zealand	Yes	No	Charity resource	2017	2	65	No
B50	https://www.caregiver.org	USA	Yes	No	Charity resource	2016	3	56.1	No
B18	https://brainxchange.ca	Canada	Yes	No	Health service resource	2012	3	62	No
G43	http://www.cerebralfunctionunit.co.uk	UK	Yes	No	Network for PLWD	2008	2	50.5	No
Y46.2	https://www.alzheimers.gov	USA	Yes	No	Public health resource	No	4	59.8	No
B18.2	https://socialcare.wales	UK/Wales	No	Yes	Social Care resource	2022	2	67	No
B6	https://www.scie.org.uk	UK	No	Yes	Social Care resource	2020	4	60.2	Yes
G26	https://www.dementiacarecentral.com	USA	No	Yes	Government funded resource	2019	3	52	No
B7	https://www.betterhealth.vic.gov.au	Australia	No	Yes	Public health resource	2014	3	51.9	No
G29	http://www.dementiamanagementstrategy.com	Australia	No	Yes	Health department resource	No	1	62.7	No
G16.2	https://www.alzsd.org	USA	No	No	Charity resource	2022	2	70.9	No
B4	https://www.dementiauk.org	UK	No	No	Charity resource	2021	2	48.4	No
G38	https://lewybodyresourcecenter.org	USA	No	No	Charity resource	2021	4	58.1	Yes
Y54	https://alzheimer.ca	Canada	No	No	Charity resource	2021	3	58.3	Yes
G10	https://alzheimer.ca	Canada	No	No	Charity resource	2021	3	55.8	No
B16	https://www.alz.org	USA	No	No	Charity site	2021	2	60.8	No
B38	https://dementiapathways.ie	Ireland	No	No	Public health / social care	2021	1	59.5	No

					resource				
Y24	https://www.ageuk.org.uk	UK	No	No	Charity resource	2021	1	56.9	No
G20	https://www.dementia.org.au	Australia	No	No	Charity resource	2020	3	61.9	Yes
B5	https://www.nhs.uk	UK	No	No	Public health resource	2020	2	44.8	No
Y57	https://www.dementiaallianceinternational.org	USA	No	No	Self-advocacy network	2020	1	65.2	Yes
B41	https://www.dementiafriendlychurch.org.uk	UK	No	No	Charity resource	2019	1	66	No
B14.2	http://www3.health.vic.gov.au	Australia	No	No	Public health resource	2014	4	52.1	No
G39	https://www.stroke.org.uk	UK	No	No	Charity resource	No	1	61.6	No
Y11	https://www.qpmc.co.uk	UK	No	No	Health service resource	No	1	44.8	No
G34	https://www.nidirect.gov.uk	UK (N. Ireland)	No	No	Public health resource	No	1	42.1	No

NB: Websites listed in order of how well they meet Silberg criteria, then by year of latest review, then by Discern score.

Appendix B

Two stage Strategy Analysis

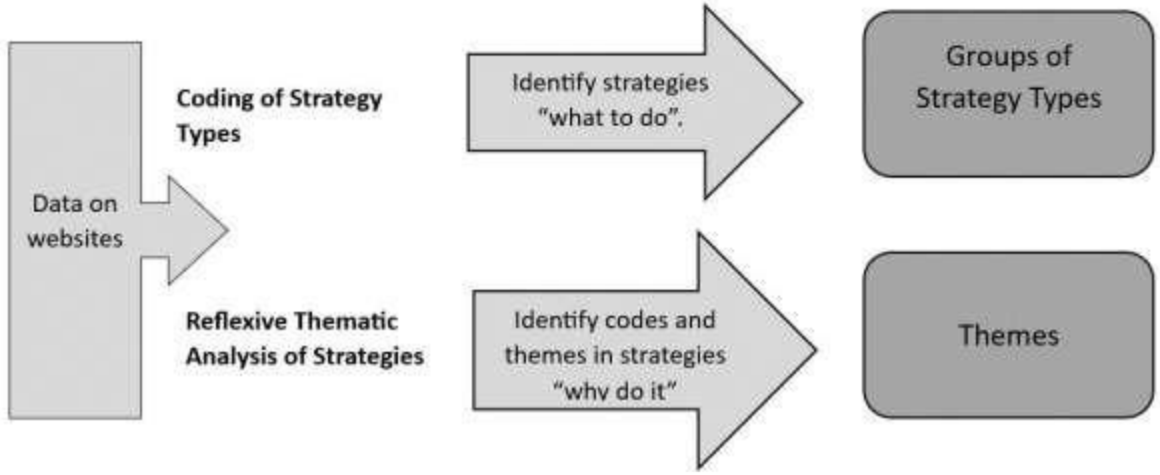

Appendix C

Final 9 themes expressing the rationale of strategies on websites, and number of strategy groups associated with each theme

Theme	Codes of strategies listed on websites	Representation across strategy type
**1. Supporting Communication Strengths**	• Communicate in a way that works to their strengths<br>• Creating conditions which support the person to communicate at their best<br>• Giving enough support for the PLWD to be successful<br>• Preparing for an interaction according to the individual's support needs<br>• Assuming communication is possible will help give a focus on remaining abilities	21/26
**2. Valuing the Interaction**	• Celebrate the interaction for its own sake<br>• A sense of connection can exist even if there is very little response<br>• Humour should always be used with respect and dignity<br>• It's ok if you don't always get the meaning, be honest, value the interaction despite it.<br>• Laughter can strengthen a relationship and reduce anxiety<br>• NVC can help gain attention and show interest<br>• Knowing when to 'give in gracefully' may take the pressure off and reduce frustration	18/26

**3. Prioritising Needs**	• Carer should look after their own needs<br>• Carer should put the PLWD first	9/26
**4. Providing Emotional Safety**	• A respectful approach helps people feel safe and supported<br>• Create a calm atmosphere to help the PLWD feel relaxed<br>• NVC can be used to create a supportive and reassuring feeling for the interaction<br>• PLWD can communicate more easily if they feel emotionally safe<br>• Avoiding unnecessary battles and 'rising above' behaviours that feel difficult<br>• Acknowledging feelings helps people feel safe to express them and move<br>• Try, above all, to be kind<br>• Even if some skills have been lost, the ability to feel emotions remains<br>• Giving the full truth can be hurtful<br>• Honesty is respectful<br>• Honesty with kindness	24/26
**5. Working Together**	• Communication is a two-way street<br>• Inclusion means creating an interaction the PLWD can join on their own terms<br>• Let me be the leader in the conversation<br>• Sharing responsibility for success and challenges<br>• Getting to the intended meaning is not always possible, despite best efforts	25/26
**6. Adapting Communication for the Situation**	• Show flexibility of communication, in the moment<br>• How you say something might depend on what you are trying to achieve	9/26
**7. Developing Carer Communication Skills**	• Carer may need to work hard to support communication effectively<br>• Carers need to be willing to adapt their communication style over time<br>• Reflect on and learn from previous experience<br>• Take responsibility for the conversation and for developing your skills<br>• Being patient with slow or repetitive speech	22/26
**8. Knowing the Individual - History and Present**	• Being genuine and honouring individuality shows intent to be supportive<br>• Consider underlying causes of communication breakdown<br>• Respecting feelings allows for a genuine relationship<br>• Seeing the person, not the dementia<br>• Understanding the PLWD's individual communication style reduces frustration<br>• Put yourself in their shoes to really understand their perspective<br>• Noticing the PLWD's reactions helps in valuing their experience and their uniqueness<br>• Recognising the PLWD’s reality builds trust and confidence in interaction	22/26
**9. Focusing on Broader Meaning**	• NVC can help convey meaning<br>• Don't give up on trying to find the meaning<br>• Creativity and compassion help interpret a person's intended meaning<br>• Feelings are real, even in not based in fact<br>• Assuming there is meaning, which may not be expressed in the words	15/26

	• The overall message may be easier to get than trying to follow every detail<br>• Too much focus on facts may highlight difficulties and feel stressful<br>• Let go of the need for everything to be correct	
Legend: PLWD = people living with dementia; NVC = non-verbal communication		

Appendix D

<u>Mapping of Strategies to Evidence</u>

**Strategy (N=100)**	**Source E1** (Egan et al, 2010)	**Source E2** (Vasse et al, 2010)	**Source E3** (Alsawy et al, 2017)	**Source E4** (Swann et al, 2018)	**Source E5** (Kindell et al, 2017)	**Source E6** (SCIE, 2013)	**Source E7** (NICE, 2018)	**Source E8** (NICE-SCIE, 2007)
**Accept their reality and join them in it**	N/A	N/A	73.8% QATSDD	N/A	Present	N/A	Present	Present
**Take your lead from the plwd**	N/A	N/A	73.81% QATSDD	N/A	Present	Present	N/A	Present
**Acknowledge their feelings and support them to express them**	N/A	N/A	N/A	N/A	N/A	N/A	Present	Present
**Look for the feelings behind the words being said**	N/A	N/A	N/A	N/A	N/A	N/A	N/A	Present
**Look for shared meaning in what plwd say or do even if it seems confusing**	N/A	N/A	N/A	N/A	Present	N/A	N/A	N/A
**Keep the person at the centre and remember what you know about them**	N/A	N/A	59.5% QATSDD	N/A	N/A	Present	Present	Present
**Learn what might be helpful from previous interactions**	N/A	N/A	73.8% QATSDD	N/A	N/A	Present	Present	Present
**Consider the culture and background of the plwd**	N/A	N/A	81.0% QATSDD	N/A	N/A	Present	N/A	Present
**Use their first language if appropriate**	N/A	N/A	N/A	N/A	N/A	Present	N/A	Present
**Use a calm friendly tone of voice**	N/A	N/A	81.0% QATSDD	N/A	N/A	N/A	N/A	Present
**Use non-verbal cues to support what you say**	N/A	N/A	N/A	N/A	N/A	Present	N/A	Present
**Use gestures and pointing or demonstration as required**	N/A	N/A	Present but not rated	N/A	N/A	Present	N/A	Present
**Use intonation to help make clear what you are saying**	N/A	N/A	N/A	N/A	N/A	N/A	N/A	Present
**Pay attention to verbal and non-verbal cues**	N/A	N/A	N/A	N/A	N/A	Present	N/A	Present

**Make eye contact**	N/A	N/A	69.1% QATSDD	N/A	N/A	N/A	N/A	N/A
**Think about your use of body language and what it represents**	N/A	SORT B 2-4 out of 9	N/A	N/A	N/A	Present	N/A	Present
**Ensure people can see your face and body language**	N/A	N/A	N/A	N/A	N/A	Present	N/A	N/A
**Learn to recognise a persons' non-verbal messages**	N/A	SORT A 6 out of 9	N/A	N/A	Present	Present	N/A	Present
**Use their reactions to gage if they are comfortable or have understood you**	N/A	N/A	73.8% QATSDD	N/A	N/A	N/A	N/A	N/A
**Look for clues in people's behaviour**	N/A	N/A	N/A	N/A	Present	Present	Present	Present
**Listen to their tone of voice**	N/A	N/A	N/A	N/A	Present	Present	N/A	Present
**Show kindness and understanding**	N/A	N/A	N/A	N/A	N/A	N/A	Present	Present
**Smile**	N/A	N/A	69.1% QATSDD	N/A	N/A	N/A	N/A	N/A
**Give friendship and support**	N/A	N/A	81.0% QATSDD	N/A	N/A	N/A	N/A	Present
**Find shared humour, laugh together where you can**	N/A	N/A	N/A	N/A	Present	N/A	N/A	N/A
**Offer reassurance and encouragement**	N/A	N/A	69.1% QATSDD	N/A	N/A	N/A	N/A	N/A
**Avoid arguing**	N/A	N/A	81.0% QATSDD	N/A	N/A	N/A	N/A	N/A
**Avoid criticizing or correcting**	SORT C	N/A	N/A	N/A	Present	N/A	N/A	N/A
**Avoid pointing out that they are repeating themselves**	N/A	N/A	N/A	Level III-2, 8 out of 15	N/A	N/A	N/A	N/A
**Accept that the plwd will make mistakes with their words sometimes**	N/A	N/A	N/A	N/A	Present	N/A	N/A	N/A
**Be positive**	N/A	N/A	81.0% QATSDD	N/A	N/A	N/A	N/A	Present
**Don't order the person around**	N/A	N/A	Present but not rated	N/A	N/A	N/A	N/A	N/A
**Avoid patronising language**	N/A	N/A	Present but not rated	N/A	N/A	N/A	N/A	N/A
**Be respectful**	N/A	N/A	59.5% QATSDD	N/A	N/A	N/A	N/A	Present
**Be honest without being hurtful**	N/A	N/A	83.3% QATSDD	N/A	N/A	N/A	N/A	N/A
**Think about how it might feel if you struggled to communicate**	N/A	N/A	N/A	N/A	N/A	Present	N/A	Present
**Be prepared for the impact that memory difficulties may have on conversation**	N/A	N/A	N/A	Level III-3, 7 out of 15	N/A	Present	Present	Present
**Learn about dementia and the changes that occur**	N/A	N/A	N/A	N/A	N/A	Present	Present	Present
**Allow for the fact that people will change**	N/A	N/A	N/A	N/A	N/A	Present	Present	N/A

**Consider font and presentation of written information**	N/A	N/A	N/A	N/A	N/A	N/A	Present	Present
**Consider hearing difficulties - speak loudly, ensure your lips can be seen, use gestures**	N/A	N/A	N/A	N/A	N/A	Present	Present	Present
**Consider medical or other reasons for difficulties**	N/A	N/A	N/A	N/A	N/A	N/A	Present	N/A
**Make sure they are wearing their glasses**	N/A	N/A	N/A	N/A	N/A	Present	N/A	Present
**Consider the best time of day for the plwd and their communication**	N/A	N/A	N/A	N/A	Present	Present	N/A	N/A
**Ensure a consistent approach to communication**	SORT C	N/A	N/A	N/A	N/A	Present	N/A	N/A
**Gently redirect to a new topic if the plwd is being very repetitive or needs distracting from something that has upset them**	N/A	N/A	69.1% QATSDD	N/A	N/A	N/A	N/A	N/A
**Initiate conversations**	N/A	Sort A 8 out of 9	N/A	N/A	N/A	N/A	N/A	N/A
**Give direction and lead the conversation when needed**	N/A	N/A	N/A	N/A	N/A	N/A	N/A	Present
**Focus on their strengths rather than what they find difficult**	N/A	N/A	N/A	N/A	Present	N/A	N/A	Present
**Give gentle prompts about names and situations as required**	N/A	N/A	69.1% QATSDD	N/A	N/A	N/A	N/A	Present
**Give tactful reminders of the topic**	N/A	N/A	N/A	N/A	N/A	N/A	N/A	Present
**Repeat key, important words**	N/A	N/A	50.0% QATSDD	N/A	N/A	N/A	N/A	N/A
**Take time to check your understanding of what they have said and the success of the communication**	N/A	N/A	N/A	N/A	Present	N/A	N/A	N/A
**Encourage communication in any way that works for the individual**	N/A	N/A	Present but not rated	N/A	Present	Present	N/A	Present
**Ask the plwd to try a different way of explaining**	N/A	N/A	50.0% QATSDD	N/A	N/A	N/A	N/A	N/A
**Make suggestions or guesses if you aren't sure what they are trying to say**	SORT C	N/A	69.1% QATSDD	N/A	Present	N/A	N/A	N/A
**Ask the plwd to point or use gestures if they can**	N/A	N/A	N/A	N/A	Present	N/A	N/A	N/A

**Ask simple clarifying questions to narrow the options if you don't understand what they are saying**	N/A	N/A	73.8% QATSDD	N/A	Present	N/A	N/A	N/A
**Give people time to think and respond**	N/A	N/A	73.8% QATSDD	N/A	Present	N/A	N/A	N/A
**Give them time to finish their sentences, don't assume**	N/A	N/A	73.8% QATSDD	N/A	N/A	N/A	N/A	N/A
**Don't have rushed conversations**	N/A	N/A	N/A	N/A	N/A	N/A	N/A	Present
**Encourage them to join in or say more**	N/A	N/A	N/A	N/A	Present	N/A	N/A	N/A
**Give them autonomy to make choices and do things independently**	N/A	N/A	N/A	N/A	N/A	Present	N/A	N/A
**Involve and include the plwd in conversations**	N/A	N/A	N/A	N/A	Present	Present	N/A	N/A
**Don't talk over the head of the plwd as if they aren't there or won't understand**	N/A	N/A	59.5% QATSDD	N/A	N/A	N/A	N/A	N/A
**Ensure your message is short and simple, broken down into chunks if necessary**	N/A	N/A	N/A	N/A	Present	N/A	N/A	N/A
**Keep sentences short and simple**	N/A	N/A	50.0% QATSDD	N/A	N/A	N/A	N/A	N/A
**Stick to one topic at a time**	SORT A	Sort A 8 out of 9	N/A	Level II, 11 out of 15	N/A	Present	N/A	N/A
**Listen even when the conversation doesn't go the way you expect**	N/A	N/A	N/A	N/A	Present	N/A	N/A	N/A
**Listen carefully and actively to what the plwd says**	N/A	N/A	73.8% QATSDD	N/A	Present	N/A	N/A	N/A
**Avoid interruptions**	N/A	N/A	50.0% QATSDD	N/A	N/A	N/A	N/A	N/A
**Reduce distracting background noise**	N/A	N/A	50.0% QATSDD	N/A	N/A	Present	N/A	N/A
**Ensure environment is relaxed and friendly and not too busy over-stimulating or intimidating**	N/A	N/A	N/A	N/A	N/A	Present	N/A	N/A
**Try to ensure people are in familiar and comfortable surroundings**	N/A	N/A	N/A	N/A	N/A	Present	N/A	N/A
**Deal with disinhibited behaviour in a matter of fact way**	N/A	N/A	N/A	N/A	Present	N/A	N/A	N/A
**Be sensitive to what difficult behaviour might really mean**	N/A	N/A	N/A	N/A	N/A	N/A	Present	Present

**Try to find time to relax and have a break**	N/A	N/A	N/A	N/A	N/A	N/A	Present	N/A
**Seek advice and support for yourself**	N/A	N/A	N/A	N/A	N/A	N/A	Present	N/A
**Look after your own health**	N/A	N/A	N/A	N/A	N/A	N/A	Present	N/A
**Speak slowly**	N/A	N/A	50.0% QATSDD	N/A	N/A	N/A	N/A	N/A
**Use the PLWD's preferred name (159)**	N/A	N/A	N/A	N/A	N/A	N/A	N/A	Present
**Move slowly**	N/A	N/A	50.0% QATSDD	N/A	N/A	N/A	N/A	N/A
**Use visual reminders**	SORT B	SORT B 2 out of 9	N/A	Level III-3, 7 out of 15	N/A	N/A	N/A	N/A
**Maintain regular routines**	N/A	N/A	N/A	Present but not rated	N/A	N/A	N/A	N/A
**Explain what you are doing before you start a task**	N/A	N/A	N/A	N/A	N/A	N/A	N/A	Present
**Check if plwd has understood you**	N/A	N/A	N/A	N/A	N/A	N/A	N/A	Present
**Be prepared to repeat, rephrase and keep trying several times**	N/A	N/A	(SEE NOTES)	N/A	N/A	N/A	Present	N/A
**Give extra contextual information to support understanding**	N/A	N/A	73.8% QATSDD	N/A	N/A	N/A	N/A	N/A
**Give visual cues to support what you are saying**	N/A	N/A	N/A	N/A	N/A	N/A	Present	Present
**If the plwd doesn't understand you try a different approach**	N/A	N/A	N/A	N/A	Present	N/A	Present	Present
**Use a card to explain individual needs or difficulties to strangers**	SORT C	N/A	N/A	Level IV, 12 out of 15	N/A	N/A	N/A	N/A
**Ask one question at a time**	N/A	N/A	50.0% QATSDD	N/A	N/A	N/A	N/A	N/A
**Use closed questions**	N/A	N/A	50.0% QATSDD	N/A	Present	N/A	N/A	N/A
**Keep questions and options simple**	N/A	N/A	N/A	N/A	N/A	N/A	Present	Present
**Support plwd to reminisce without testing them**	N/A	SORT A 5 out of 9	69.1% QATSDD	Level III-2, 7 out of 15	N/A	Present	N/A	Present
**Help people engage in activities they enjoy**	N/A	N/A	N/A	N/A	N/A	Present	Present	N/A
**Find things around you to support a topic for conversation**	N/A	N/A	N/A	Level IV, 10 out of 15	N/A	N/A	N/A	N/A
**Find activities to share and support expression**	SORT B	N/A	73.8% QATSDD	Level III-2, 11 out of 15	N/A	Present	N/A	Present
**Encourage the use of multiple senses**	N/A	SORT B 6 out of 9	N/A	N/A	N/A	N/A	Present	N/A
**Use reassuring touch**	N/A	N/A	69.1% QATSDD	N/A	N/A	N/A	N/A	N/A

Evidence rating systems for systematic review sources

- SORT (Strength of Recommendation Taxonomy, Ebell et al 2004) system. Strength of evidence ranged between SORT A (consistent and good quality, patient-oriented evidence) and SORT C (consensus, usual practice, opinion, disease-oriented evidence, or case series for studies of diagnosis, treatment, prevention, or screening). Used by source 1 (Egan et al, 2010) and source 2 (Vasse et al, 2010).
- QATSDD (Quality Assessment Tool for Studies with Diverse Designs, Sirriyeh et al 2012), a mixed methods quality assessment tool which gives a percentage score for strength of evidence – ratings ranged from 50% to 83.3% (some strategies mentioned in the paper were not given a strength rating). Used by source 3 (Alsawy et al, 2017).
- National Health and Medical Research Council (NHMRC) (2009) evidence levels. Ratings fell within the range Level II (a randomised controlled trial) to Level IV (a case series with either post-test or pre-test/post-test outcomes). Use by source 4 (Swann et al, 2018).
- Criteria for reviewing conversation analysis (Parry and Land, 2013). Used by source 5 (Kindell et al, 2017).

References

Alsawy, S., Mansell, W., McEvoy, P., & Tai, S. (2017). What is good communication for people living with dementia? A mixed-methods systematic review. International Psychogeriatrics, *29*, 11, 1785–1800. doi.org/10.1017/S1041610217001429

Ebell, M. H.et al (2004). Strength of Recommendation Taxonomy (SORT): A patient-centered approach to grading evidence in the medical literature. Journal of the American Board of Family Practice, 17, 1, 59–67. doi.org/10.3122/jabfm.17.1.59

Egan, M., Bérubé, D., Eve Racine, G., Leonard, C., & Rochon, E. (2010). Methods to Enhance Verbal Communication between Individuals with Alzheimer's Disease and Their Formal and Informal Caregivers: A Systematic Review. Research International Journal of Alzheimer's Disease, 2010, 1-12. doi.org/10.4061/2010/906818

Kindell, J., Keady, J., Sage, K., & Wilkinson, R. (2017). Everyday conversation in dementia: a review of the literature to inform research and practice. International Journal of Language and Communication Disorders, 52, 4, 392–406. doi.org/10.1111/1460-6984.12298

National Health and Medical Research Council (NHMRC). (2009).NHMRC additional levels of evidence and grades for recommendations for developers of guidelines. Retrieved from https://www.nhmrc.gov.au/_files_nhmrc/file/guidelines/developers/nhmrc_levels_grades_evidence_120423.pdf

Sirriyeh, R., Lawton, R., Gardner, P., & Armitage, G. (2012). Reviewing studies with diverse designs: The development and evaluation of a new tool. Journal of Evaluation in Clinical Practice, 18, 4, 746–752. doi.org/10.1111/j.1365-2753.2011.01662.x

Swan, K., Hopper, M., Wenke, R., Jackson, C., Till, T., & Conway, E. (2018). Speech-language pathologist interventions for communication in moderate–severe dementia: A systematic review. American Journal of Speech-Language Pathology, 27, 2, 836–852. doi.org/10.1044/2017_AJSLP-17-0043

Vasse, E., Vernooij-Dassen, M., Spijker, A., Rikkert, M. O., & Koopmans, R. (2010). A systematic review of communication strategies for people with dementia in residential and nursing homes. International Psychogeriatrics, *22*, 2, 189–200. doi.org/10.1017/S1041610209990615
